# Evaluation of the cytotoxic effects of ophthalmic solutions containing benzalkonium chloride on corneal epithelium using an organotypic 3-D model

**DOI:** 10.1186/1471-2415-9-5

**Published:** 2009-07-28

**Authors:** Su Khoh-Reiter, Bart A Jessen

**Affiliations:** 1 Drug Safety Research and Development, Pfizer Inc, 10646 Science Center Drive, San Diego, CA 92121, USA

## Abstract

**Background:**

Benzalkonium chloride (BAC) is a common preservative used in ophthalmic solutions. The aim of this study was to compare the cytotoxic effects of BAC-containing ophthalmic solutions with a BAC-free ophthalmic solution using an organotypic 3-dimensional (3-D) corneal epithelial model and to determine the effects of latanoprost ophthalmic solution and its BAC-containing vehicle on corneal thickness in a monkey model.

**Methods:**

The cytotoxicity of commercially available BAC-containing ophthalmic formulations of latanoprost (0.02% BAC) and olopatadine (0.01% BAC) was compared to that of BAC-free travoprost and saline in a corneal organotypic 3-D model using incubation times of 10 and 25 minutes. To compare the extent of differentiation of 3-D corneal cultures to monolayer transformed human corneal epithelial (HCE-T) cell cultures, expression levels (mRNA and protein) of the corneal markers epidermal growth factor receptor, transglutaminase 1 and involucrin were quantified. Finally, latanoprost ophthalmic solution or its vehicle was administered at suprapharmacologic doses (two 30 μL drops twice daily in 1 eye for 1 year) in monkey eyes, and corneal pachymetry was performed at baseline and at weeks 4, 13, 26 and 52.

**Results:**

In the 3-D corneal epithelial culture assays, there were no significant differences in cytotoxicity between the BAC-containing latanoprost and olopatadine ophthalmic solutions and BAC-free travoprost ophthalmic solution at either the 10- or 25-minute time points. The 3-D cultures expressed higher levels of corneal epithelial markers than the HCE-T monolayers, indicating a greater degree of differentiation. There were no significant differences between the corneal thickness of monkey eyes treated with latanoprost ophthalmic solution or its vehicle (both containing 0.02% BAC) and untreated eyes.

**Conclusion:**

The lack of cytotoxicity demonstrated in 3-D corneal cultures and in monkey studies suggests that the levels of BAC contained in ophthalmic solutions are not likely to cause significant direct toxicity to epithelium of otherwise normal corneas.

## Background

Unpreserved multiuse ocular solutions are at an increased risk of microbial contamination [1] and can potentially cause catastrophic consequences similar to those reported with inadequately preserved contact lens solutions [2]. Because of its established efficacy compared to other agents, the most common preservative used in topical ocular solutions is benzalkonium chloride (BAC) [3]. Despite its long-standing clinical safety record supported by extensive use in many pharmaceuticals and cosmetics, the presence of BAC in topical solutions has been implicated as a potential cause of corneal irritation, particularly in situations where the corneal surface is compromised such as that resulting from dry eye syndrome [4,5]. Ocular hypotensive agents containing BAC have been reported to have greater toxicity than BAC-free solutions when evaluated in a transformed human corneal epithelial (HCE-T) monolayer cell culture model [6]. However, this system does not adequately duplicate the stratified nature of corneal epithelium, which is better reflected in organotypic 3-dimensional (3-D) culturing conditions. Toxicity associated with BAC-containing ophthalmic solutions has also been demonstrated in rabbits [7-9], which are known to be considerably more sensitive to corneal irritation [10] and to have a longer duration of irritation [11] than humans. The increased sensitivity may be due in part to rabbits having an extremely low blink rate [12] and thinner corneas [10] compared to humans.

Because of the limitations of the HCE-T monolayer and rabbit models, the use of other *in vitro *model systems has been explored. One such model is a commercially available 3-D construct (SkinEthic Laboratories, Nice, France) in which immortalized human corneal epithelial cells are grown on an inert permeable polycarbonate insert and cultivated at the air-liquid interface in a defined medium [13-15]. These constructs have been shown to be histologically and ultrastructurally similar to the stratified cellular organization of human corneal epithelium and to express corneal-specific keratins [14]. Studies have shown a high reliability and concordance of the 3-D constructs with *in vivo *models in predicting irritancy of test compounds [13,15,16].

The aim of this study was to compare the corneal cytotoxic effects of the commercially available ophthalmic solutions of latanoprost (containing 0.02% BAC) and travoprost (containing the preservative sofZia and no BAC), both ocular hypotensive agents, and olopatadine ophthalmic solution, an antihistamine (containing 0.01% BAC), using the 3-D model. Olopatadine was selected as a test solution, due to its BAC content, to control for the mechanism of the pharmacologically active ingredient and its usage in the treatment of ocular irritation associated with allergies. Furthermore, the expression levels of basal and suprabasal corneal epithelial markers were compared between the 3-D and monolayer models to determine the relative degrees of differentiation. Finally, we report the findings of a 1-year study evaluating the effects of latanoprost ophthalmic solution containing 0.02% BAC on corneal thickness in monkeys receiving a daily suprapharmacologic dose of latanoprost.

## Methods

### Cell cultures

Corneal epithelial 3-D culture inserts and the maintenance medium (MCDB 153, 5 μg/mL insulin, 1.5 mM CaCl_2_, 25 μg/mL gentamicin) were purchased from SkinEthic Laboratories. The inserts were allowed to equilibrate overnight in 6-well plates with 1 mL of the maintenance medium at 37°C and 5% CO_2 _and then were transferred into 300 μL of maintenance medium in a 24-well plate prior to testing.

The HCE-T cell line 10.014 pRSV-T was obtained from the American Type Culture Collection (Manassas, VA) and grown to approximately 80% confluence in flasks coated with collagen (PuroCol^®^; Inamed Biomaterials, Fremont, CA), fibronectin, bovine serum albumin (Fraction V) and hydrocortisone in a defined keratinocyte serum-free medium (Invitrogen, Carlsbad, CA) according to the vendor's protocol.

### In vitro cytotoxicity assay

To demonstrate the sensitivity of the 3-D cultures, BAC at concentrations of 0.005%, 0.01%, 0.025%, 0.05%, 0.075% and 0.1% and saponin at concentrations of 0.01%, 0.05%, 0.1%, 0.25% and 0.5% were incubated in duplicate inserts for 60 minutes at 37°C. Statistical analysis was not performed due to the limited number of replicates. Once the sensitivity of the cultures was determined, 50 μL of test solutions was added to the 3-D cultures and incubated at 37°C for 10 or 25 minutes. Test solutions included latanoprost (Xalatan^®^) ophthalmic solution 0.005% (with 0.02% BAC: Pfizer Inc, New York, NY), olopatadine (Patanol^®^) ophthalmic solution 0.1% (with 0.01% BAC: Alcon, Fort Worth, TX), travoprost (Travatan Z^®^) ophthalmic solution 0.004% (with sofZia: Alcon, Fort Worth, TX), 5% saponin (Sigma-Aldrich, St. Louis, MO), 70% methanol and normal saline (0.9% NaCl solution; Baxter, Deerfield, IL). Rinsed inserts were then transferred to a new 24-well plate containing 300 μL of 0.5 mg/mL MTT (3-{4, 5-dimethylthiazol-2yl} 2, 5-diphenyltetrazolium bromide; Sigma-Aldrich, St. Louis, MO) in maintenance medium and incubated for 3 hours at 37°C. After removal of the medium, isopropanol was added at room temperature and plates were placed on an orbital shaker for 90 minutes after which the absorbance of the extracts was measured at 575 nm (SpectraMAX 190: Molecular Devices, Sunnyvale, CA). Conditions were tested in triplicate wells in three separate experiments. The absorbance of each sample was normalized to the mean saline control absorbance for each experiment. Data from all three experiments (N = 9 samples) were combined for statistical analysis using a 1-way analysis of variance.

### Real-time quantitative reverse transcriptase-polymerase chain reaction assay

The 3-D cultures were rinsed with Dulbecco's phosphate-buffered saline (DPBS) without calcium and magnesium (Invitrogen), incubated with dispase at 37°C for 2 minutes and detached from the insert by gentle scraping with forceps. The HCE-T monolayers were rinsed with DPBS prior to harvesting in 700 uL of RLT buffer. RNA was extracted using the RNeasy kit (Qiagen, Valencia, CA), and RNA quality (as determined by 18s and 28s ribosomal RNA integrity) was assessed using the Lab-on-a-Chip Nanochip (Agilent, Santa Clara, CA).

To perform the real-time quantitative reverse transcriptase-polymerase chain reaction assay, 1 μg of RNA was used as a template to generate cDNA with the QuantiTect Reverse Transcription kit (Qiagen). The relative expression of epidermal growth factor receptor (EGFR) was determined using 5 μM primers (Integrated DNA Technologies, San Diego, CA) and fluorescent SYBR Green dye (QuantiTect SYBR Green PCR kit, Qiagen) incorporation using the LightCycler^® ^2.0 System (Roche Applied Sciences, Indianapolis, IN). The amplification was performed in 20 μL reactions with optimized cycling and annealing conditions. The specific primers and probes for involucrin and transglutaminase 1 (TG1) were designed with Roche's online Universal Probe Library (UPL) system's Assay Design Center. Each 20 μL reaction mixture contained 5 μL cDNA, 4 μL 5× qPCR Master Mix, 2 μL each primer (5 μM) (Integrated DNA Technologies), 0.4 μL probe (10 μM) (Roche Applied Sciences) and 6.6 μL of water. The samples were then loaded onto a 96-well plate and amplified with an initial denaturation protocol of 95°C for 10 minutes followed by 45 cycles of 95°C for 10 seconds, 60°C for 30 seconds and 40°C for 30 seconds. The expression of target genes was normalized to β-actin. Standard curves were generated for each assay run. The primers for EGFR were 5'-AATGCTTTCACAACATTTGC-3' (forward) and 5'-ACAGGGCACACACAGATTAG-3' (reverse). The primers for involucrin were 5'-GAAAGCAGAAAACCCAGAGC-3' (forward) and 5'-TAGCTGCTGATCCCTTTGTG-3' (reverse) (Roche UPL probe number 2). The primers for TG1 were 5'-CAAGAGACTAGCAGTGG-3' (forward) and 5'-AGGCCATTCTTGATGGACTC-3' (reverse; UPL probe number 84). Statistical comparisons were performed using a 1-tailed Student's t test.

### Western blot assay

Prior to lysis, the HCE-T monolayers were rinsed twice in cold phosphate-buffered saline to remove the residual medium; the maintenance medium was removed from the 3-D inserts and the inserts were frozen at -80°C for 20 minutes. All samples were lysed in 100 μL of radioimmune precipitation assay buffer with 1× HALT protease inhibitors (Pierce, Rockland, IL). Lysates were centrifuged at 16,000 × g for 10 minutes at 4°C and the supernatants were collected; protein concentrations were determined using the bicinconinic acid protein assay (Pierce). Approximately 40 μg of protein was heated to 95°C for 5 minutes in NuPAGE 10× sample-reducing agent/LDS 4× Sample Buffer (Invitrogen), electrophoretically separated with a NuPAGE 4–12% Bis-Tris gel (Invitrogen) and transferred to a 0.2 μm nitrocellulose membrane. After blocking in fluorescent blocking buffer (Rockland Immunochemicals, Gilbertsville, PA) for 1 hour at room temperature, the membrane was incubated with primary antibodies overnight at 4°C. Primary antibodies against corneal markers included rabbit anti-human EGFR (1:1000; 100-401-149: Rockland Immunochemicals), rabbit anti-human involucrin (1:50; BT-651: Biomedical Technologies Inc., Stoughton, MA) and mouse anti-human TG1 (1:50; BT-621: Biomedical Technologies Inc.). To verify consistency in protein loading, the membranes were incubated with respective species-specific β-actin antibody (rabbit, catalog number 600-401-886, Rockland Immunochemicals; or mouse, catalog number sc-47778, Santa Cruz Biotechnology, Inc., Santa Cruz, CA). After three washes in 25 mM Tris/150 mM NaCl/0.1% Tween, the membranes were probed with appropriate secondary infrared antibodies (donkey anti-rabbit IgG, 611-730-127, or rabbit anti-mouse IgG1, 610-430-040: Rockland Immunochemicals) for 1 hour at room temperature before visualizing on the Odyssey infrared scanner (Li-Cor Biosciences, Lincoln, NE).

### In vivo monkey pachymetry study

A chronic topical ocular toxicity study was conducted to support the initial registration of latanoprost ophthalmic solution. Cynomolgus monkeys (5/sex/group) were treated for 1 year with topical ocular administration of suprapharmacologic doses of latanoprost in a concentrated formulation specifically prepared for toxicity testing (0.08%; 2–30 μL drops in 1 eye twice daily for a total dose of 100 μg/day) or an equal volume of vehicle containing 0.02% BAC. Study monkeys received latanoprost in the right eye and its vehicle (containing 0.02% BAC) in the left eye (2–30 μL drops twice daily for both treatments) while control monkeys received no treatment in the right eye and vehicle in the left eye. Corneal thickness (pachymetry) was measured with an Ophthanasonic Pachymeter (Teknar, St. Louis, MO) prior to dosing and at weeks 4, 13, 26 and 52 of the dosing period. Mean pachymetry values for the left vehicle-treated eye were compared with those of the right eye (latanoprost-treated or untreated). A Student's t test was used to determine the statistical significance of the differences between treatment conditions. Other data collected in the study included, but were not limited to, ocular histopathology, ophthalmic examinations and clinical signs. These studies were conducted in accordance with the Association for Research in Vision and Ophthalmology Statement for the Use of Animals in Ophthalmic and Visual Research and were in compliance with Good Laboratory Practices.

## Results

### In vitro cytotoxicity assays

**Figure 1:**
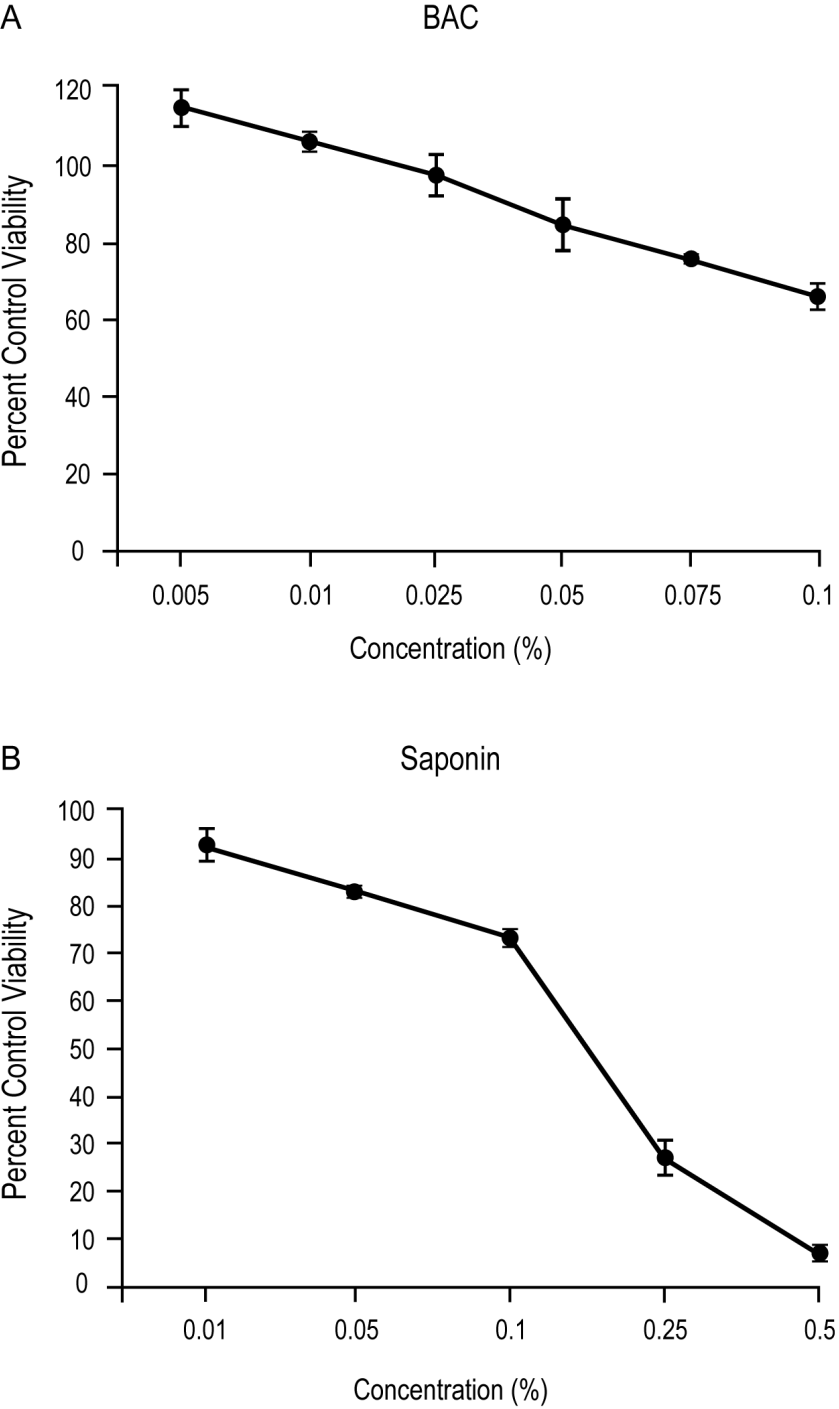
**Cytotoxicity of benzalkonium chloride (BAC) and saponin in 3-D corneal epithelial cultures**. The viability of the 3-D corneal inserts was determined after treatment with BAC (A) or saponin (B) for 60 minutes at 37°C. Data represent mean percent (± range) of the saline control.

When the sensitivity of 3-D cultures to BAC-induced cytotoxicity was evaluated by incubating the inserts with a range of concentrations of BAC for 60 minutes (Figure 1A), BAC concentrations comparable to those found in commercial ocular solutions (<0.025%) showed no differences in viability relative to the saline control. Statistical analysis was not performed due to the limited number of replicates. At the highest BAC concentration tested (0.1%), the viability was reduced to 66%. The positive control (saponin at concentrations ranging from 0.01% to 0.5%) displayed extensive cytotoxicity (Figure 1B), with viability as low as 6% relative to the saline control at the high dose of 0.5%. These results established that the 3-D cultures demonstrated dose-dependent toxicity of BAC.

**Figure 2:**
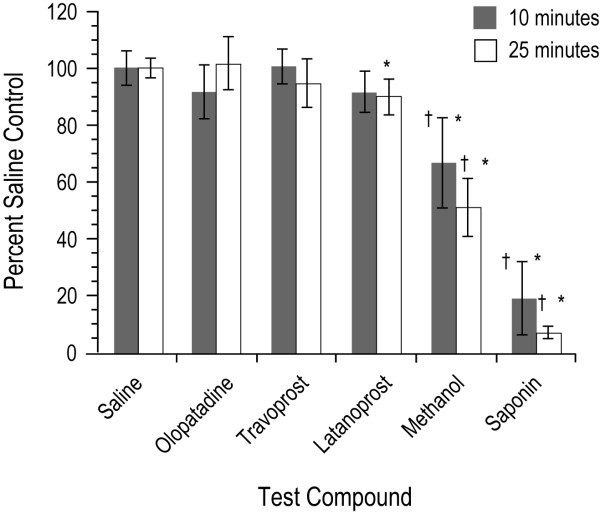
**The effects of the ophthalmic solutions on 3-D human corneal epithelial cell viability reported as mean percent (± standard deviation) of saline control**. Methanol (70%) and saponin (5%) were positive controls and saline was a negative control. *p < 0.05 versus saline control. ^†^p < 0.05 versus travoprost.

The commercially available formulations of latanoprost, olopatadine and travoprost were evaluated using incubation times of 10 or 25 minutes (Figure 2). With the 10-minute incubation time, only the positive controls (methanol and saponin) had a significant decrease in viability compared to the saline control (p < 0.05). With the 25-minute incubation time the difference in viability with latanoprost ophthalmic solution was significantly lower than with the saline control (p < 0.05); however, there were no significant differences in viability with latanoprost or olopatadine when compared with BAC-free travoprost (p > 0.05).

### Corneal epithelial marker expression

**Figure 3:**
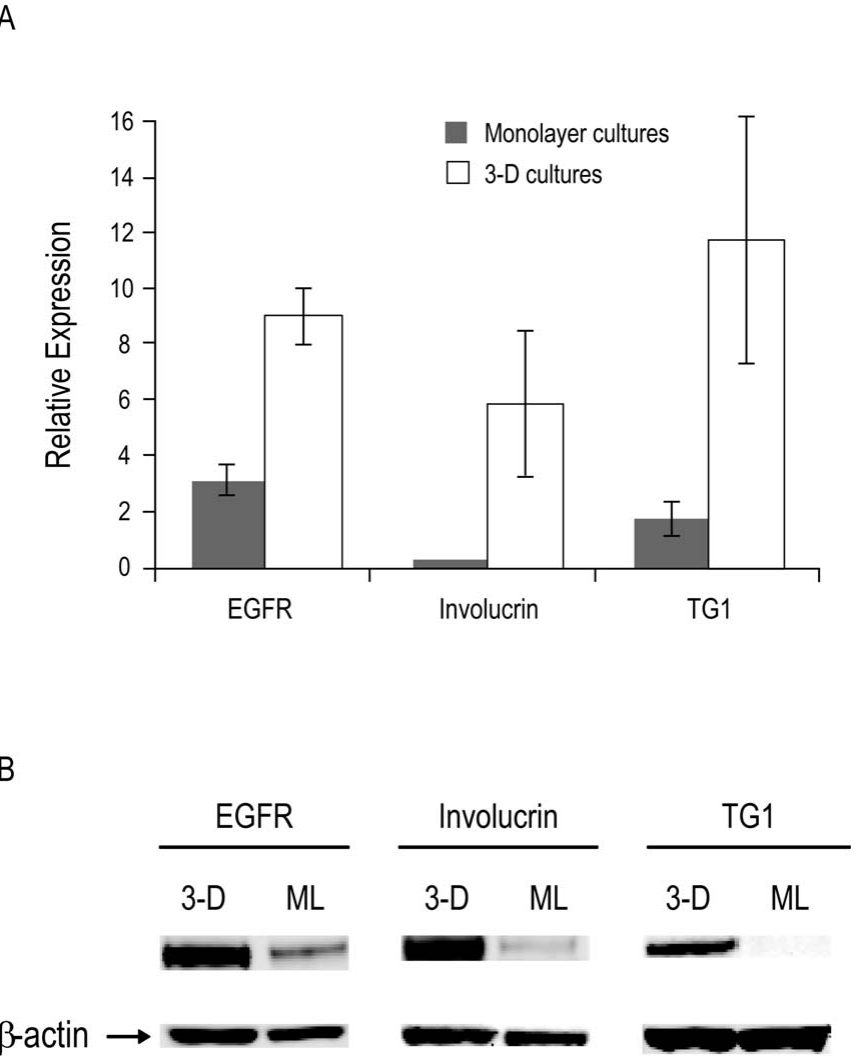
**Expression of corneal epithelial gene markers**. (A) The relative mRNA expression levels of epidermal growth factor receptor (EGFR), involucrin and transglutaminase 1 (TG1) in 3-D and transformed human corneal epithelial (HCE-T) monolayer (ML) cultures as measured by real-time quantitative reverse transcriptase-polymerase chain reaction assay, normalized to β-actin expression. (B) The relative protein expression levels of EGFR, involucrin and TG1 as determined by Western blot assay; β-actin was used as a loading control.

The expression levels of three corneal epithelial markers (EGFR, involucrin and TG1) were evaluated in both the 3-D cell cultures and the HCE-T monolayer cultures. After normalizing for β-actin expression, mRNA levels for all three corneal epithelial markers were expressed at significantly higher levels in the 3-D cultures than in the monolayer cultures (Figure 3A). Specifically, when compared to the monolayer cultures, the expression of mRNA levels in the 3-D cultures were 3-fold (p < 0.001), 45-fold (p < 0.05) and 7-fold (p < 0.05) greater for EGFR, involucrin and TG1 mRNA levels, respectively. The relative levels of protein expression of the corneal epithelial markers as determined by Western blot assay were consistent with the mRNA expression, with the 3-D model demonstrating higher levels of EGFR, involucrin and TG1 protein than the monolayer cultures (Figure 3B).

### Monkey pachymetry studies

**Figure 4:**
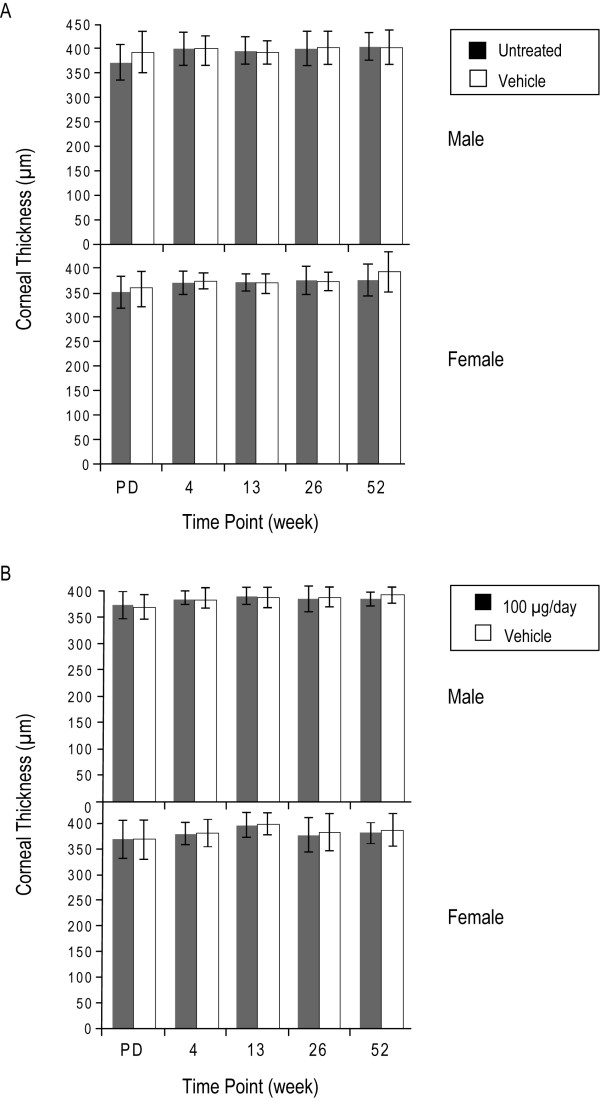
**Corneal thickness measurements from 1-year monkey studies**. (A) Effect of topical administration of latanoprost vehicle containing 0.2 mg/mL (0.02%) benzalkonium chloride on corneal thickness in monkeys. p > 0.4 for vehicle-treated versus untreated eyes in both male and female monkeys at all time points. PD = predose. (B) Effect of the topical administration of suprapharmacologic doses of latanoprost (100 μg/day) on corneal thickness in monkeys. p > 0.5 for the vehicle-treated versus latanoprost-treated eyes in both male and female monkeys at all time points. PD = predose.

In the 1-year monkey pachymetry studies, there was no statistically significant difference (p > 0.4) in mean corneal thickness between the eyes treated with BAC-containing vehicle and untreated eyes at any of the four time points (Figure 4A). Similarly, in monkeys that were administered suprapharmacologic doses of latanoprost and vehicle (containing 0.02% BAC) of equal volume in the other eye, there was no statistical difference (p > 0.5) in the mean corneal thickness between the two treatments at any point throughout the 52 weeks (Figure 4B). No histological effects as assessed by light microscopy or clinical signs of irritation were noted in the study (data not shown).

## Discussion

Organotypic 3-D corneal epithelial cultures approximate corneal epithelium, forming stratified layers approximately 60 μm thick and expressing corneal-specific keratins [14]. The layers consist of four to six cells, with flattened superficial cells and several layers of wing cells; ultrastructurally, the cells demonstrate surface microvilli, tight junctions, numerous intercellular interdigitations and desmosomes and a basement membrane [15]. The 3-D cultures also have been shown to develop similar barrier properties to corneal epithelium as measured by transepithelial electrical resistance [13,17].

The assays presented herein using 3-D corneal epithelial cultures demonstrated no significant differences in cytotoxicity between the BAC-containing latanoprost and olopatadine ophthalmic solutions and BAC-free travoprost ophthalmic solution at either of the time points (10 and 25 minutes) tested. Our results are in contrast to a study demonstrating latanoprost toxicity in monolayer cultures of HCE-T cells [6]. The same group reported that up to 70% of the cells grown under these conditions died in 5 minutes in their control, which was treated with culture medium alone [18]. However, HCE-T monolayer cultures have been found to exhibit cytotoxicity when exposed to concentrations of detergents well below those affecting the viability of 3-D cultures [17]. Furthermore, an ophthalmic solution that was nontoxic in rabbit corneas and 3-D cultures was shown to cause breakage of cell junctions and vacuolization in HCE-T monolayers [19]. These studies suggest that HCE-T monolayer cultures are not appropriate for evaluating the cytotoxicity of ophthalmic compounds.

The difference in cytotoxicity between the HCE-T monolayers and the 3-D cultures may reflect the extent of corneal differentiation. We demonstrated that 3-D cultures express significantly higher mRNA and protein levels of three corneal epithelial markers (EGFR, involucrin and TG1) compared to HCE-T monolayers. EGFR expression has been demonstrated in the basal layers of human corneal epithelium [20]. Involucrin has been observed in suprabasal layers as is consistent with a role in differentiation [20]. TG1 has been identified in the corneal epithelium, mostly in suprabasal cells and in the uppermost keratocyte layer, where it is found in association with fibrillin-containing microfibrils [21]. The relatively higher expression level of EGFR in the monolayer system compared to the other markers may reflect its expression in the basal, less differentiated cell types.

The 3-D corneal cultures have been validated against the Draize rabbit irritation assay [15,16]. In a multiple test-site study, the 3-D model gave an 80% overall concordance with rabbit irritancy data with 100% sensitivity [16]. A range in exposure times (10, 20, 30 and 60 minutes) was investigated, with a 10-minute exposure found to be the most predictive of 24-hour rabbit irritancy scores. Cytotoxicity as measured by MTT reduction correlated highly with both histopathological evaluation of the cultures and the *in vivo *irritancy scores [16].

The duration of exposure of 3-D cultures to test compounds in other studies has varied widely among researchers, with some using 5-minute exposures [13,17] and others using 20- and 60-minute exposures [19]. In one study demonstrating toxicity of BAC in 3-D cultures at concentrations as low as 0.005%, exposure times were an extraordinarily long 6 and 24 hours [22]. In our assays, exposure to BAC concentrations of 0.02% for 60 minutes demonstrated no toxicity relative to the saline control. Moreover, these durations far exceed those expected in human eyes, for which mean ocular surface residence times have been shown to be as low as 2.9 seconds [23].

In addition to the studies in the 3-D corneal cultures, we conducted a 1-year corneal pachymetry study in monkeys that demonstrated no effects of suprapharmacologic doses (67 times the recommended clinical dose of latanoprost and 4 times the associated dose of BAC) on corneal thickness. Corneal pachymetry has been shown to be a predictive measure of ocular irritation resulting in edema in the corneal epithelium [24]. The absence of corneal thickness changes, irritation or histological changes implies a lack of a clinically significant or biologically functional effect.

The results of our assays demonstrated no significant differences in toxicity between commercially available BAC-containing latanoprost and olopatadine ophthalmic solutions and BAC-free travoprost ophthalmic solution using 3-D corneal epithelial cultures as an *in vitro *model of corneal cytotoxicity. Although 3-D cultures have some regenerative capacity, they lack certain components of the ocular surface that are involved in healing of corneal tissues, including the tear film, the corneal stroma and endothelium and the limbal vascular supply, which likely increases their sensitivity to toxic injury [17]. Consistent with the results of our study, Townley and Reilly [25] recently reported that when patients experiencing ocular dryness and irritation while receiving latanoprost with BAC for at least 1 month were randomized to receive latanoprost in one eye and travoprost without BAC in the other eye, significantly more corneal staining was found in the eyes receiving travoprost (p = 0.025). In addition, the eyes receiving BAC-free travoprost showed a trend toward more dryness.

## Conclusion

The lack of cytotoxicity demonstrated here using 3-D corneal cultures combined with the monkey pachymetry data suggest that the levels of BAC in BAC-containing ophthalmic solutions do not likely cause significant direct toxicity to the epithelium of otherwise normal corneas. Data supporting the safety of BAC are contained in documentation submitted to regulatory agencies in the support of both prescription and nonprescription (over-the-counter) products in ophthalmic, otic and nasal preparations for several decades. The results of this study support the present acceptable safety profile for such agents.

## Competing interests

SKR and BAJ are employees of and own shares of Pfizer Inc, the sponsor of these studies.

## Authors' contributions

SKR and BAJ were both involved with the design, conduct, collection of the data, management of the in vitro studies, analysis of the data, and preparation, review and approval of the manuscript. Both authors read and approved the final version of the manuscript.

## Pre-publication history

The pre-publication history for this paper can be accessed here:


